# Sexual behavior and vulnerability to HIV infection among seasonal migrant laborers in Metema district, northwest Ethiopia: a cross-sectional study

**DOI:** 10.1186/s12889-015-1468-0

**Published:** 2015-02-11

**Authors:** Kassa Tiruneh, Belaynew Wasie, Hector Gonzalez

**Affiliations:** Ethiopia Network for HIV/AIDS Treatment, Care and Support (ENHAT-CS) Program Management Sciences for Health, Bahirdar, Ethiopia; Bahirdar University, Department of Epidemiology and Public Health, Bahirdar, Ethiopia; American Century University, Santa Fe, New Mexico USA

**Keywords:** HIV/AIDS, Sexual Behaviors, Vulnerability, Seasonal workers, Northwest Ethiopia

## Abstract

**Background:**

Poor socio-economic conditions fuel seasonal migration of adult males from Northwestern Ethiopia, but behavioral and other migration-related changes increase their vulnerability to HIV/AIDS. This study examined risky sexual behaviors and associated factors that may lead to increased HIV infection vulnerability among migrant laborers in Metema District, Ethiopia.

**Methods:**

A community-based cross-sectional study was conducted from July 8–18, 2013 at farms with migrant laborers. We enrolled 756 participants through multistage random sampling. Data were collected using structured questionnaires and analyzed using EPI Info7; bivariate and multivariate logistic regression analyses were performed using SPSS.

**Results:**

582 (77%) migrant workers had sexual intercourse in their lifetime. 68% (397/582) reported non-marital sexual intercourse in the preceding six months. Of these, 74% reported sexual intercourse with commercial sex workers, 49% reported having transactional sex, 49% reported unprotected sexual intercourse with CSWs, 69% reported multiple sexual partners in the preceding six months (mean = 2.9 ± 0.7). Being aged between 20–29 (AOR = 2.15, 95% CI: 1.16, 3.99) and 30 years or older (AOR = 2.51, 95% CI: 1.1, 5.71), receipt of HIV prevention information in the preceding six months (AOR = 1.74, 95% CI: 1.15, 2.63), and staying longer on the farm (AOR = 2.74, 95% CI: 1.46, 5.14) were factors significantly associated with condom use at last non-marital sexual intercourse. Respondents aged ≤19, not receiving HIV information in the preceding six months, or staying on the farm for ≤2 months were less likely to have used condoms at their last non-marital sexual intercourse. Moreover, having daily income above USD 5.00 (AOR = 2.24, 95% CI: 1.14, 4.41), paying for most recent sexual intercourse (AOR = 2.22, 95% CI: 1.36, 3.61), and drinking alcohol during last sexual intercourse (AOR = 1.69, 95% CI: 1.01, 2.83) were significantly associated with having multiple (≥2) sexual partners during the preceding six months.

**Conclusions:**

Seasonal laborers commonly exhibit risky sexual behaviors likely to increase their vulnerability to HIV infection. Unprotected and multiple sex partners in these populations pose transmission risks to seasonal laborers and onward to their wives and future sexual partners. The findings support the need for targeted HIV prevention campaigns designed for seasonal workers and their sexual partners.

## Background

Ethiopia has nearly 800,000 people living with Human Immunodeficiency Virus (HIV). Adult HIV prevalence is estimated at 1.5%, with highest rates in urban areas (4.2%) and for females (1.9%). Amhara region has the second largest number of HIV infected persons in Ethiopia [1].

Poor socio-economic conditions have fueled seasonal migration of adult males from Northwestern Ethiopia. Around 200,000 - 300,000 migrants leave home annually to find seasonal farm work in Metema, Quara, and West Armacheho Districts, returning home in three- to six-month intervals [2,3]. Migrant laborers experience a high prevalence of HIV and sexually transmitted infections (STIs) and a low level of condom use [4].

Migration is a primary cause of behavior change, as migrants are exposed to behaviors and norms that differ from those in their place of origin [5-8]. Migrants are away from their spouses, families, and homes for extended periods, which can lead to isolation and anxiety. They have limited familial and social support networks, and limited pressure from the social norms that govern sexual behavior. They are forced into physically demanding jobs and poor housing and living conditions, and have limited access to health care and health information. These factors may put migrant workers at risk of HIV infection, though many of these factors are not necessarily specific to labor migrants and are shared with other vulnerable populations [9]. When seasonal migrant workers leave their familiar environment with traditional norms and values, the anonymity of being a foreigner might increase risky sexual activities such as multiple casual sexual partners, engaging in sex with commercial sex workers (CSWs), and alcohol abuse [5,7,10,11]. Such behavioral and other migration-related changes make seasonal migrant workers vulnerable to human immunodeficiency virus/acquired immune deficiency syndrome (HIV/AIDS) and other serious health risks [12].

Population mobility and migration especially, contribute to the phenomenon of concurrent sexual partnerships, which is arguably one of the main drivers of the HIV epidemic. Because migrants and mobile workers are separated from their permanent partners, they are more inclined to engage in short- or long-term sexual relations with other partners [13]. Migrant workers are likely to engage in unprotected sexual activities with high-risk populations such as CSWs, which place them at high risk for acquiring HIV or other STIs. Studies in India revealed that 30% of migrant workers have higher rates of risky sexual behaviors such as non-marital sexual intercourse at the place of destination [14]. Other studies in South Africa (31.4%), North Carolina (46%), and California (30%) indicated that migrant workers living apart from their wives are likely to engage in higher rates of multiple and commercial sex [13,15,16]. Studies in India (25%), South Africa (33%), and Croatia (44.7%) revealed that condom use is less practiced among seasonal migrant workers having sexual contact with any casual or commercial sexual partners [17-19]. Another study in South Africa revealed that condom use among migrant farm workers at last risky sex is 53.6% [13].

**Figure 1:**
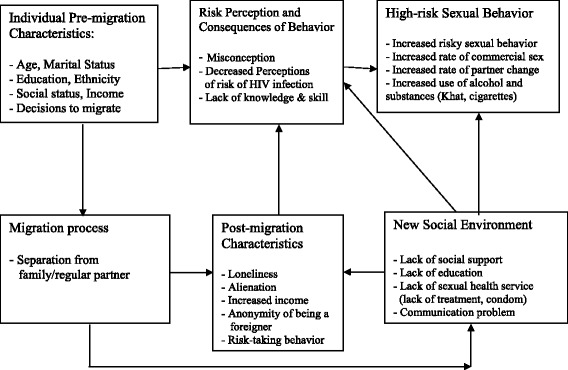
**Conceptual framework, the influence of migration on sexual behavior.** (Brockerhoff & Biddlecom, [6]; cited in Brummer, 2002).

Migrants’ risk of HIV infection is largely determined by their sexual behavior; high-risk sexual behavior among migrants is usually attributed to changes as a result of migration. As clearly indicated by Brockerhoff & Biddlecom [6] and cited in Brummer [5] there are three factors related to migrants’ sexual behavior: pre-migration individual characteristics, changes in individual characteristics due to migration, and exposure to new physical and social environments [5]. These factors play a part in the construction of certain perceptions of risk, and eventually have an effect on the actual sexual behavior of migrants. The following conceptual model (Figure 1) shows the influence of migration on risky sexual behavior.

As indicated in this conceptual framework, individual pre-migration characteristics influence the decision to migrate and migrants’ perceptions of risk of HIV infection. The migration process itself changes some of the individual characteristics into post-migration individual characteristics. A new social environment can result in a lack of social support, which has been linked to risk-taking behavior. Moreover, migrants experience many problems living in a new environment. This may influence the migrant workers’ mental and physical health [5].

The association between migration, mobility, and infection with HIV has been documented almost since the beginning of the AIDS epidemic [20,21]. Migration/mobility of at-risk individuals, particularly the relocation of individuals or frequent visits to other areas for economic opportunity has been viewed as a strong co-factor in rising HIV prevalence [22].

From an epidemiological perspective, migrant populations are often perceived to act as “bridge populations”, forming a link between high and low prevalence groups [5,6,8,23-25]. Migration facilitates the rapid spread of HIV and other infectious diseases [26]. Migrants not only display more risky sexual behaviors, but serve as a bridge population for spreading HIV from destination areas to their place of origin [27]. It is widely believed that migrant men acquire infections at the migrant’s receiving destination and continue to have sexual contact with their spouse and regular sexual partners upon returning to their native place. Hence, subsequent sexual activity with low-risk populations, like their spouses and regular sexual partners, transmits infection to populations that would otherwise be unlikely to acquire HIV or other STI [11,28].

Because of stigma and discrimination associated with HIV and STI, and due to the reason that returnee migrants may not want to consider themselves at risk and thus may not want to get screened, they are less likely to receive screening for possible HIV infections. This ultimately increases the risk of STI for their spouses and regular sexual partners, and contributes to more rapid transmission of HIV/AIDS at the migrants’ places of origin and throughout the region/country [7,29]. The risk is enhanced by the low frequency of consistent condom use among returnee migrants having sexual contact with their spouses and regular sexual partners as a result of poor condom promotion, education, and utilization efforts [5,6,14,30]. In addition, migrant workers are less willing to use condoms during sex with their spouses and regular sexual partners; they may feel that condoms are not appealing because of connotations of multiple partnerships [31].

With the number of migrants who are at risk of HIV infection continuing to rise, and with the epidemic spreading to rural areas throughout their places of origin, these issues are all the more timely and important.

### Justification of the study

In the Amhara region of Ethiopia there is an expansion of large farms; rapid migration of young people towards farms in the region is high. Most studies on HIV transmission in Ethiopia have focused primarily on the main cities in which HIV/AIDS has already spread. However, no studies have investigated the mobility patterns of male seasonal migrant laborers, the changes in sexual behaviors accompanying migration, and the implications of seasonal migration for the spread of HIV infection. Moreover, HIV risk among migrant workers has not been studied despite the potential for migrant workers to rapidly transmit HIV to other populations. Adequate data is not available for population specific planning of interventions in the region. Hence, understanding the sexual behaviors and associated factors that lead to increased HIV infection vulnerability among seasonal migrant laborers is critical to designing targeted HIV prevention campaigns for seasonal migrant workers and their sexual partners, including spouses in both destinations and places of origin, and to curbing the spread of HIV/AIDS in the community [12,32].

### Objective of the study

The current study was conducted to examine risky sexual behaviors and associated factors that lead to increased HIV infection vulnerability among seasonal migrant laborers in Metema District, Northwest Ethiopia. The findings of this study will aid in the development of targeted HIV prevention and awareness programs.

## Methods

### Study design

A community-based, cross-sectional study design was used to gather information on sexual behavior and vulnerability of seasonal migrant laborers.

### Study setting

The study was conducted from July 8–18, 2013 at farms in Metema, Northwest Ethiopia. Metema district is located in North Gondar Province of the Amhara Regional State. It is 158 km from Gondar and 338 km from Bahirdar (the capital of Amhara). According to the 2007 population census estimate, Metema has a population of 119,050 permanent residents. Among these, 63,433 are males and 55,617 are females [33]. There are over 80,000 seasonal migrant workers travelling annually to Metema for temporary labor; they return to their original residence after three to six months of stay every year.

The 2009 Ante Natal Care (ANC) sentinel surveillance survey report of the Ethiopian Ministry of Health found an elevated prevalence of HIV infection in Metema hospital (7.5%), while the National (Ethiopia) and Regional (Amhara) HIV prevalence was 1.5% and 1.6% respectively [34]. In Metema district there are one district hospital, five health centers, twenty-three health posts, and three satellite clinics [35].

### Study population

The study population comprised seasonal male migrant laborers who were on-site during the study period. There are 174 farms in Delelo numbers 1, 2, and 3 and Mertread, where the migrant workers usually get deployed. On average, the number of migrant laborers deployed annually in Delelo 1, 2, and 3 and Mertread are 25,000, 20,000, 25,000, and 10,000, respectively [36]. Data were collected at fourteen randomly selected farms over the four main sites.

### Sampling

There are 154 farms in Delelo and 20 farms in Mertread. The average number of seasonal migrant laborers in each farm is estimated to be 460, which makes the total number of migrant workers 80,040 on the 174 farms. In the first stage of sampling, primary sample units (PSUs) were formed by segmenting the farms so that each PSU would include approximately 460 male migrant workers. A total of 174 PSUs were formed; of these, 14 farms/PSUs (two from Mertread, and twelve from Delelo), referred to as “study sites”, were selected by lottery. Subsequently, the required sample from each study site was determined by systematic random sampling using a list provided by hiring farm owners.

The sample size was calculated using a population proportion formula based on providing a 95% confidence interval with a 5% margin of error. Sample size was determined based on the following assumptions: we fixed the confidence level (1- α) at 95%, and power (1-β) at 80%. With a design effect of 2, the total sample size needed was 658. Assuming a 15% non-response rate, a total of 756 participants were recruited for the quantitative survey.

### Data collection tools and procedures

Pre-tested structured questionnaires were used to collect data from respondents. Fifty-five questions were developed to assess socio-demographic characteristics, migration related behaviors, knowledge and perception of HIV risk, and risky sexual behaviors of seasonal migrant workers. The questionnaires were developed after review of relevant literature, and adapted partly from the HIV/AIDS Behavioral Surveillance Survey [37] (Family Health International), UNGASS indicators, and other published journal articles [38,39]. The questionnaires were slightly modified for clarity of terms and order of questions.

Twelve Nurses and Health Officers were recruited for data collection. One-day intensive training was given for data collectors. The training focused on understanding the meaning of each question, the need to obtain an informed consent, keeping confidentiality of the information they gathered, and quality of data as well as the techniques of presenting the questions for participants in an understandable manner. Individual face-to-face interviews were conducted for an hour at farms. During data collection, frequent checkups were made by the principal investigators to ensure the completeness and consistency of the data. The returned questionnaires were checked for completeness by the investigators.

### Ethical considerations

Procedures of this study were reviewed and approved by, and ethical clearance and letter of permission were obtained from, the Amhara Regional State Health Bureau Institutional Review Board (IRB). Participants of the study were introduced to the purpose of the study and the importance of their participation in the study. They were informed that participation was completely voluntary and what they would tell us was completely confidential. Informed oral consent was obtained from each participant involved in the study before the interview.

### Study variables

Dependent variables were 1) condom use at last sex, and 2) multiple partners (≥2) in the past 6 months. The independent variables included socio-demographic variables, income, duration of stay at farm, knowledge of HIV, perception of HIV risk, paying for sex, alcohol use at last sex, smoking cigarettes, and chewing Khat.

### Operational definitions

#### Risky sexual behavior

If the respondents have multiple (≥2) sexual partners in 6 months, or do not use condoms in last sexual intercourse, or have transactional sex.

#### Vulnerability to HIV infection

The lack of power of individual and communities to minimize their risk of exposure to HIV infection, and once infected, to receive adequate care and support. Vulnerability to HIV was considered when encountering multiple partners without consistent condom use in the preceding 6 months. Thus, we considered a seasonal migrant worker as vulnerable when he had two or more extra-marital sexual relationships and was unable to use condom in every sexual intercourse.

### Data quality control

The quality of data was maintained through the use of standard and/or pre-tested/structured questionnaires, appropriate selection and training of supervisors and data collectors, proper supervision during data collection. Moreover, the data were double-entered and checked for consistency.

### Data analysis

Data collected from respondents were cleaned, categorized, and coded on a well-drafted coding sheet, and entered in the computer with EPI Info version 7. Analyses were performed using SPSS version 20. During the analysis, frequencies of the study variables were determined; odds ratios (OR) and 95% confidence intervals (CIs) were calculated to determine associations of selected variables with the outcome variable. For some variables, medians and interquartile ranges (IQR) are listed. Descriptive, bivariate, and multivariate logistic regression analyses were implemented to explore and determine the association between predictors and outcome variables, and to control confounders. A p-value of <0.05 and 95% CI that did not include 1 [unity] were considered to be statistically significant.

## Results

### Socio-Demographic characteristics of seasonal migrant laborers

A total of 756 individuals participated in the interview with a response rate of 100%. A majority, 747 (98.8%) of respondents were from Amhara region. Four hundred sixty-nine (62.9%) respondents were between ages 20 and 29 years, with a median age of 22 years (IQR) 20–25]; 188 (24.9%) respondents were ages 19 and under. Two hundred seventy-two (36.0%) respondents were unable to read and write, 257 (34%) had primary education, and 135 (17.8%) had secondary and above education. About 498 (65.9%) respondents were never married, and 258 (34.1%) were married. Orthodox Christian 736 (97.3%) was the dominant religion of the respondents (Table 1).

**Table 1:** **Socio-Demographic Characteristics of Seasonal Migrant Laborers at Metema Farm Areas, Northwest Ethiopia, 2013 (n = 756)**

**Characteristic**	**Number of respondents**	**Percent**
Farm site		
Delelo	648	85.7
Mertread	108	14.3
Age (median, 22; IQR, 20–25)		
19 and below	188	24.9
20-29	469	62.0
30+	99	13.1
Ethnicity		
Amhara	743	98.3
Others	13	1.7
Place of Origin (Region)		
Amhara	747	98.8
Others	9	1.2
Province		
North Gondar	392	52.5
South Gondar	223	29.8
Others	132	17.7
Religion		
Muslim	18	2.4
Orthodox	736	97.3
Protestant	2	0.3
Marital status		
Have spouse/regular partner	258	34.1
Have no spouse/regular partner	498	65.9
Education		
Unable to read and write	272	36.0
Read and write	92	12.2
Primary	257	34.0
Secondary	119	15.7
Diploma and above	16	2.1

### Migration-related characteristics

Two-thirds (499; 66%) of seasonal workers had migrated to work at Metema farms for at least the second time. Most (96.6%) seasonal farmworkers migrated to work at age 15 or later; the median age at first migration was 19 (IQR 17–22). Further, 523 (69.2%) of respondents stayed 2 months and fewer in the farm area; 150 (19.8%) stayed 3 to 4 months. The daily income for a seasonal farm worker ranged from USD 2.00 to11.00 with a median daily income of USD 3.80. Most (64%) migrant farmworkers reported they had used alcohol in the preceding 6 months. Smoking cigarettes and chewing Khat were reported by 110 (14.6%) and 57 (7.5%) respondents respectively.

### Knowledge of prevention and perception of HIV risk of seasonal migrant laborers

Most (753; 99.6%) respondents had heard of HIV and AIDS. However, only 434 (57.4%) respondents received information about HIV/AIDS in the preceding 6 months; of those, 416 (95.9%) received HIV information before arriving at the farm. Most (750; 99.2%) respondents knew that HIV can be transmitted from person to person. When further asked about their knowledge of HIV transmission, many (86.6%) respondents mentioned HIV could be transmitted by puncture with infected needles and sharp materials. Moreover, participants reported HIV could be transmitted by sexual intercourse (77%), unprotected sex (36.8%), and infected blood contact (22.4%). However, only 7.8% of the respondents reported HIV could be transmitted by having sex with multiple sex partners, and most respondents were unsure about transmission of HIV through pregnancy, delivery, and breast feeding. Further, when asked how seasonal migrant laborers were protecting themselves form HIV infection, the most frequently mentioned HIV prevention methods described by most respondents was having sex with only one sexual partner (50.8%). Another HIV prevention method reported by 46% of respondents was using condoms consistently. Though not using contaminated needles/sharp materials was reported by 41.4% of the respondents, abstaining from pre- and extramarital sex was acknowledged by a similar number (39.4%) of respondents as one of the preferred HIV prevention methods.

In this study, although there was not a clear understanding of every aspect of HIV/AIDS, 571 (75.5%) respondents perceived that HIV/AIDS is a very high/high-risk disease. However, 185 (24.4%) seasonal migrant laborers did not appear to be aware of their risk of HIV infection, and believed that their risk of getting HIV infection was very small.

### Risky sexual behaviors of seasonal migrant laborers

Most (582; 77%) migrant workers had had sexual intercourse in their life time. Of these, 579 (99.6%) had sex at age 15 or later. Age at first sexual intercourse for migrant workers ranged between 13 and 29 years. The mean age at first intercourse was 18.8 years (±2.3). Most (397/582) respondents reported non-marital sexual intercourse in the preceding six months. Of these, 293 (74%) reported sexual intercourse with commercial sex workers (CSWs). Further, 69% (273/397) reported multiple sexual partners in the preceding six months (mean = 2.9, SD ± 0.7), and 49% reported having transactional sex.

Condom use was uncommon and inconsistent among seasonal migrant farm workers. A large proportion (57.6%) of respondents reported they never use a condom during any sex episodes. Although 247/582 (42.4%) of the seasonal migrant laborers had ever used condoms during any sexual intercourse, only 132 (53.4%) of them used condoms consistently. While condom usage was mentioned as a means of protecting oneself against HIV/AIDS by 46% of respondents, 195/397 (49.1%) of the respondents reported unprotected sexual intercourse during their most recent sexual contact with non-marital sexual partners. The main reason given by 85.6% of sexually active migrant workers for not using condoms was their belief that condoms reduce sexual pleasure [prefer to have sex skin to skin] and that they were at low risk of contracting HIV. Further, 296/397 (74.6%) of respondents who had had non-marital sex in the previous 6 months had consumed alcohol at their last sex with CSWs. Moreover, 283/582 (48.6%) of the respondents who had ever had sex had sex with a paid partner in the preceding 6 months (Table 2).

**Table 2:** **Risky Sexual Behaviors of Seasonal Migrant Laborers at Metema Farm Areas, Northwest Ethiopia, 2013 (n = 756)**

**Characteristic**	**Number of respondents**	**Percent**
Never had sex	174	23.0
Had ever sex	582	77.0
Age at first sex (mean ± s.d., 18.8 ± 2.3) (n = 582)		
• Before age 15	3	0.5
• At age 15 or later	579	99.5
Ever had non-marital sex in the last 6 months		
Yes	397	68.2
Ever consumed alcohol during the last non-marital sex (n = 397)		
Yes	296	74.6
Number of non-marital sex partners in the past 6 months (mean ± s.d. 2.9 ± 0.7) (n = 397)		
1	124	31.2
2	183	46.1
3+	90	22.7
Type of non-marital sex partners		
Commercial sex workers	293	73.8
Girlfriend/causal partner	104	26.2
Ever paid money for sex, in the past 6 months (n = 582)		
Yes	283	48.6
Ever used a condom (n = 582)		
Yes	247	42.4
Consistency of condom use (n = 247)		
Use condom every time/consistently	132	53.4
Use condom most times	34	13.8
Use condom sometimes	81	32.8
Used condom at last non-marital sex (=397)		
Yes	202	50.9
Consistency of condom use during non-marital sex (n = 202)		
Use condoms every time/consistently	128	90.2
Use condoms most times	22	2.9
Use condoms sometimes	52	6.9
Reason for not using condoms at non-marital sex (n = 195)		
Prefer skin to skin	167	85.6
I trust her	16	8.2
She may think I am HIV-positive	5	2.6
Condoms not available	7	3.6
Use condom at each marital sex episode (n = 258)		
Yes	17	6.6
Ever discuss HIV/AIDS with wife/regular partner (n = 258)		
Yes	156	60.5
No	102	39.5
Reason for not discussing HIV/AIDS with spouse		
Uncomfortable /difficult to discuss	79	81
It is not something to discuss	23	19

### Factors associated with condom use at last non-marital sexual intercourse

Multivariate logistic regression analyses revealed age, receipt of HIV prevention information, and staying longer on the farm were factors significantly associated with condom use at last non-marital sexual intercourse. Respondents ages 20–29 (AOR = 2.15, 95% CI: 1.16, 3.99) and ages 30 and over (AOR = 2.51, 95% CI: 1.1, 5.71) were more likely to use condoms at their last non-marital sexual intercourse than those ages 19 and under. Respondents who had ever received information on HIV/AIDS in the preceding six months were 1.74 times more likely to use condoms at their last non-marital sexual intercourse than those who had not received (AOR = 1.74, 95% CI: 1.15,2.63). Moreover, those seasonal migrant farmworkers who stayed in the farm area five to six months were 2.74 times more likely to use condoms at their last non-marital sexual intercourse than those who stayed for two months or fewer (AOR = 2.74, 95% CI: 1.46,5.14) (Table 3).

**Table 3:** **Factors Associated With Condom Use at Last Non-Marital Sexual Intercourse Among Seasonal Migrant Laborers at Metema Farm Areas, Northwest Ethiopia, 2013**

**Variable**	**Condom use**		**Crude OR**	**Adjusted OR**	**P-value**
	**Yes**	**No**	**(95% CI)**	**(95% CI)**	
Age (years)					0.037
19 and under	18	38	1:00	1:00	
20-29	156	137	2.40(1.31,4.40)	2.15(1.16,3.99)	0.015
30+	28	20	2.95(1.32,6.59)	2.51(1.1, 5.71)	0.029
Duration of stay at farm, since came here					0.004
2 months or less	110	137	1:00	1:00	
3 - 4 months	51	41	1.55(0.95,2.50)		
5 - 6 months	41	17	3.00(1.62,5.58)	2.74(1.46,5.14)	0.002
Information on HIV/AIDS in the last 6 months					
Yes	101	68	1.87(1.25,2.80)	1.74(1.15,2.63)	0.009
No	101	127	1:00	1:00	
Ever paid money for sex in the past 6 months					
Yes	53	69	0.65(0.42,0.99)		
No	149	126	1:00		

***Note:*** *Forward stepwise binary logistic regression analysis indicating the net effect of the explanatory variables on the outcome variables.*

### Factors associated with having multiple sexual partners during the preceding 6 months

Multivariate logistic regression analyses indicated that daily income, paying for sex in the preceding 6 months, and consuming alcohol during the last sex were significantly associated with having multiple sexual partners during the preceding 6 months.

Respondents who earned a daily income above USD 5.00 were 2.24 times more likely to have multiple sexual partners during the preceding 6 months than those having a daily income below USD 5.00 (AOR = 2.24, 95% CI: 1.14,4.41). Similarly, seasonal farmworkers who paid for sex were 2.22 times more likely to have multiple sexual partners during the last 6 months than those who did not pay for sex in the last 6 months (AOR = 2.22, 95% CI: 1.36,3.61). Moreover, seasonal workers who consumed alcohol at their last sexual intercourse were 1.69 times more likely to have multiple sexual partners during the preceding 6 months than those who did not drink alcohol during their last sex (AOR = 1.69, 95% CI: 1.01,2.83) (Table 4).

**Table 4:** **Factors Associated With Having Multiple Sex Partners During the Last 6 Months Among Seasonal Migrant Laborers at Metema Farm Areas, Northwest Ethiopia, 2013**

**Variable**	**Multiple**		**Crude OR**	**Adjusted OR**	**p-value**
	**sex partners**		**(95% CI)**	**(95% CI)**	
	**Yes**	**No**			
Daily income					
USD 3.00 and below	82	54	1:00	1:00	
3.00 3.80	68	24	1.87 (1.05, 3.33)		
3.80- 5.00	69	30	1.51 (0.87, 2.62)		
Above 5.00	54	16	2.22 (1.15, 4.28)	2.24 (1.14,4.41)	0.019
Paid for sex in the past 6 months					
Yes	208	67	2.72 (1.74,4.27)	2.22 (1.36,3.61)	0.001
No	65	57	1:00	1.00	
Consumed alcohol during last sex					
Yes	217	79	2.21 (1.38, 3.53)	1.69 (1.01,2.83)	0.046
No	56	45	1:00	1.00	
Ever chew Khat					
Yes	36	8	2.20 (0.99, 4.89)		
No	237	116	1:00		

***Note:*** *Backward stepwise binary logistic regression analysis indicating the net effect of the explanatory variables on the outcome variables.*

## Discussion

The results of the current study demonstrate that nearly 96% of seasonal migrant laborers in this study had not received recent HIV/AIDS-related education at their place of destination. There may be several possible reasons why seasonal migrant workers had not received recent HIV/AIDS-related education: their mobility, lack of participation in community conversation services, and lack of preventive programs in the new environment are potential explanations.

Higher rates of risky sexual behaviors and practices were common among respondents. Nearly 68% of sexually active seasonal migrant workers reported non-marital sexual intercourse in the preceding 6 months, which is double that found in India (30%) [10,14]. Further, engaging in sex with CSWs was common (74%) while at the farm. This finding is higher than in studies of migrant workers living apart from their wives in North Carolina (46%) [11] and California (30%) [12] in the U.S. The current study also found nearly half (49%) of respondents had transactional sex, and 69% of sexually active participants had more than one non-marital sexual partner. This exceeds the finding of a study in South Africa (36%) [13] and Jimma and Deredawa, Ethiopia [40].

Daily income, paying for sex, and drinking alcohol during the last sex were factors associated with encountering multiple sexual partners. Workers with a higher income were more likely to have multiple sexual partners during the preceding 6 months than men who earn less; this finding is consistent with that of India [17]. Similarly, seasonal farmworkers who paid for sex were more likely to have multiple sexual partners. This finding is also consistent with that of India [Ibid]. As income increases, it is possible that the ability to pay for sex increases, making workers more likely to engage in sexual intercourse with multiple sex partners. Additionally, the availability of disposable income could create opportunities for expanding sexual networks.

Seasonal workers who consumed alcohol in the preceding 6 months were more likely to have multiple sexual partners than those who did not consume alcohol; consistent with findings from a similar study in India [Ibid], South Africa [13], and Butajira, Ethiopian [41]. The findings may be explained, in part, by increased opportunity for casual partners at beer houses. Moreover, sex after drinking alcohol is more likely unprotected because alcohol decreases self-control and sexual negotiation skills of young adults. Further, alcohol use drives visits to sex workers and unsafe sex practices [42].

These risky behaviors likely stem from the changes occurring when seasonal migrant laborers leave their familiar environment, with traditional norms and values, for a new environment in which the anonymity of being a foreigner might increase risky sexual activities [5,18,43,44]. Separation from family and regular sexual partners and the breakdown of traditional family units might result in high-risk sexual behavior such as alcohol use. In addition to this, the seasonal migrant workers’ individual characteristics (such as age, marital status, education and use of Alcohol) or social environment (such as peer influence, lack of social support while away from family, lack of information on HIV/AIDS, increased income) may lead to risky sexual activity and initiation of multiple sexual partners [5,15,45]. This approach of sexuality can result in high-risk sexual behavior that leads to increased vulnerability to HIV [46].

Uncommon and inconsistent condom use was reported among seasonal farm workers in Metema farm areas. More than half (57.6%) of sexually active respondents reported they usually did not use a condom during any sex episodes. In addition, 49% of the respondents did not use condoms during their recent non-marital sexual intercourse. Only 51% of the respondents used condoms during their recent non-marital sexual intercourse. This percentage was higher when compared with studies done in India and South Africa [14,17,18] where only 33%, 25%, and 38% male migrant workers reported condom use during sex with CSWs, and further exceeds that reported in the 2005 HIV/AIDS Behavioral Surveillance Survey (BSS) in Ethiopia, where condom use among youths, migrants, and mobile workers in Amhara Region was lowest (24%). This finding was similar to the percentages of migrant farm workers in South Africa (53.6%) [13] and Croatia (44.7%) [19], which indicates that farm workers at Metema are not using condoms consistently. This low percentage of condom use suggests that a higher rate of HIV infection and other sexually transmitted infection may exist among seasonal migrant laborers at Metema District. The reason could be the respondents’ low perception of HIV risk as well as their belief that condoms reduce sexual pleasure [12]. Additionally alcohol consumption appears to have impaired decision-making, promoting risky sexual activities, such as multiple sexual partnership. However, respondents who had received appropriate information on HIV/AIDS in the preceding six months were more likely to use condoms at their last non-marital sexual intercourse. This finding is consistent with a study conducted in Kenya [6].

Interestingly, those who had been on the farm longer were more likely to use a condom than those who had arrived more recently. Possible explanations for this phenomenon are that individuals residing on the farms longer may have more interaction with peers and be more aware of appropriate HIV prevention methods and sources of condoms. This finding is consistent with that of South Africa [47]. It may be that newer arrivals lack social support or are affected by the new social environment as they lack information on availability of HIV prevention methods including sources of condoms. Moreover, those ages 20–29 and ages 30 and over were more likely to use condoms at their last non-marital sexual intercourse than those ages 19 and under, although condom use was uncommon and inconsistent among seasonal migrant workers.

The poor results of promotion efforts to encourage consistent condom use within regular partnerships highlight this as one of the major challenges in condom promotion. In sum, the findings of this study demonstrate the relationship between labor migration and migrant workers’ risky sexual behaviors.

### Strengths and limitations of the study

The study focused on a group of people who are highly vulnerable to HIV and AIDS where little is known about them. However, there is the possibility of social desirability bias due to the sensitive nature of the subject matter. Some respondents did not provide actual information about their sexual behavior and practice. Hence, under-reporting of sexual partners may have occurred due to some forms of interview bias. In addition, the study is limited only on the individual level; the broader context (population-level factors) for HIV vulnerability is not acknowledged.

## Conclusions

In the response to AIDS in Ethiopia, it is important to address specific groups, communities, or locations. Seasonal migrant workers or mobile populations are high HIV-risk groups. The factors or processes that contribute to the vulnerability of seasonal migrant workers to HIV infection are diverse, complex, and not fully understood. However, the study documented that a great proportion of migrant farm workers reported having higher risky sexual behaviors and practices, including engaging in sexual intercourse with commercial sex workers, transactional sex, having inconsistent and low condom use, having multiple sex partners that increase the seasonal migrant laborers’ vulnerability to HIV infection.

The findings of this study suggest that a well-organized information, education, and communication effort is warranted to effect behavioral change. Hence, several recommendations can be made based on the major findings and conclusions of this study. In particular, targeted HIV/AIDS prevention and treatment interventions like condom access, education, and utilization programs should be in place at migrant workers’ destinations and farm sites. On the other hand, to prevent transmission of HIV to spouses and regular sexual partners, promotion of condom use and HIV testing at departure and return of migrant workers are recommended. In addition, research is needed to further understand the prevalence of HIV and determinants of seasonal migrant workers’ vulnerability to HIV infection. Furthermore, research that focuses on broader context (population-level factors) for HIV vulnerability need to be conducted.
